# Prevention and inhibition of post-harvest browning in longkong pericarp using *Prunus Persica* resin coating during ambient storage

**DOI:** 10.1371/journal.pone.0323416

**Published:** 2025-05-12

**Authors:** Narin Charoenphun, Somwang Lekjing, Karthikeyan Venkatachalam

**Affiliations:** 1 Faculty of Innovative Agriculture, Fisheries and Food, Prince of Songkla University, Surat Thani Campus, Makham Tia, Mueang, Surat Thani, Thailand; 2 Faculty of Science and Arts, Burapha University, Chanthaburi Campus, Chanthaburi, Thailand; National Institute of Food Technology Entrepreneurship and Management, INDIA

## Abstract

**Background:**

Longkong fruit is highly perishable due to rapid pericarp browning, limiting its post-harvest shelf life to less than 7 days under ambient conditions.

**Objectives:**

This study evaluated the efficacy of *Prunus persica* resin (PPR) coatings at 1%, 2%, 3%, and 4% concentrations in preserving fruit quality over 14 days of ambient storage, with assessments conducted at two-day intervals.

**Methods:**

Longkong fruits were treated with PPR coatings and stored under ambient conditions. Parameters including pericarp browning, decay rate, moisture loss, color retention, biochemical properties, and enzymatic activities were analyzed.

**Results:**

PPR-coated samples exhibited superior performance compared to control samples, with higher PPR concentrations effectively mitigating pericarp browning, decay rate, and moisture loss while maintaining fruit color and biochemical properties. Control fruits became unacceptable by day 8, exhibiting a browning index exceeding 4.51 and a decay rate of 82.56%, whereas fruits treated with higher PPR concentrations (>3%) maintained lower browning indices (<3.47) and decay rates (<58.99%) by day 14. Color retention was significantly enhanced in 4% PPR-treated fruits, which maintained lightness, redness, and yellowness values, while untreated fruits exhibited severe discoloration. Moisture loss in untreated fruits reached 35.31% by day 8, whereas 4% PPR-treated fruits limited moisture loss to 20% throughout the storage period. Additionally, high concentrations of PPR coatings preserved higher total phenolic content, flavonoid levels, and antioxidant activities. PPR-coated longkong fruit effectively suppressed the activity of browning-related enzymes such as polyphenol oxidase, peroxidase, and cinnamate-4-hydroxylase, alongside reductions in membrane-degrading enzymes lipoxygenase and phospholipase D.

**Conclusion:**

These findings indicate that PPR coatings, particularly at 4% concentration, effectively extend longkong fruit shelf life for up to 14 days, providing a natural, biodegradable post-harvest solution. This approach holds significant potential for reducing food waste, supporting sustainable agricultural practices, and enhancing marketability across longkong fruit supply chains, particularly benefiting small-scale farmers in tropical regions.

## 1. Introduction

Longkong (*Aglaia dookkoo* Griff) is a widely consumed and economically significant fruit in Southeast Asia, particularly in Thailand. It consists of 5–6 pale, translucent flesh segments encased in a dark yellowish, slightly leathery pericarp, with 1–5 inedible seeds. While the pericarp and seeds are not consumed, they have been traditionally used in both contemporary and indigenous medicine due to their bioactive compounds and health-enhancing properties [1]. Longkong fruit’s distinct flavor, nutritional benefits, and rising market demand have driven its increasing commercialization. However, its short post-harvest shelf life, typically lasting only 3–7 days under ambient conditions, significantly limits its export potential and market expansion [2]. One of the primary challenges in longkong storage is pericarp deterioration, which results from high moisture content, microbial decay, enzymatic browning, and physical damage during handling. Studies indicate that longkong pericarp can lose up to 30–40% of its market value within a week due to browning and shrinkage, severely impacting both local and international trade [3]. Among the various deterioration factors, enzymatic browning is the major contributor to post-harvest discoloration, predominantly catalyzed by polyphenol oxidase (PPO) and peroxidase (POD), which become active upon pericarp injury [4]. The synergistic action of PPO, POD, and pericarp phenolic compounds rapidly accelerate browning, reducing fruit quality during storage [3]. Additionally, cinnamate-4-hydroxylase (C4H), a cytochrome P450 enzyme in the phenylpropanoid pathway, is one of the key contributors for producing phenolic compounds [5]. Browning in longkong fruit is further exacerbated by cell membrane-degrading enzymes, including lipoxygenase (LOX), lipase, and phospholipase D (PLD). LOX facilitates the oxidation of polyunsaturated fatty acids, generating reactive oxygen species (ROS) that induce oxidative stress, accelerating pericarp deterioration [6]. Comparative studies show that LOX activity in longkong pericarp increases rapidly within the first 48 hours of storage, coinciding with the rapid onset of browning [7–8]. Lipase catalyzes lipid hydrolysis, breaking down triglycerides and phospholipids, leading to membrane instability and further exposure of phenolic substrates to browning enzymes [6]. The collective impact of these oxidative processes severely compromises the visual and textural appeal of longkong, making effective post-harvest interventions crucial.

A variety of post-harvest treatments have been explored to mitigate pericarp browning and extend the shelf life of longkong. Common approaches include modified atmospheric packaging, phytohormone coatings, chitosan coatings, low-temperature storage, and organic acid applications and however, these methods have notable limitations [1]. In contrast, chemical-based treatments, though effective, raise concerns about toxic residues, regulatory restrictions, and consumer health risks. Chitosan and phytohormone coatings require specialized processing, increasing production costs. On the other hand, low-temperature storage leads to severe chilling injury in longkong and incurs high energy costs, making it an impractical solution for long-term preservation [3]. Organic acid treatments including citric acid and oxalic acid, can reduce PPO activity but may cause surface damage or alter longkong flavor when applied at high concentrations [9]. The development of a safe, cost-effective, and sustainable approach is essential for preserving longkong during storage and transport. Edible coatings provide a biodegradable and eco-friendly alternative, reducing dependence on synthetic plastics, minimizing environmental pollution, and widely preserving fruits, vegetables, meat, dairy products, and ready-to-eat foods [10–11]. Recent studies highlight the potential of plant-based resin coatings, particularly *Prunus persica* resin (PPR), as a promising natural preservative for fresh produce [12]. PPR is a biodegradable polysaccharide that forms a protective barrier, restricting oxygen exposure to inhibit enzymatic browning while stabilizing phenolic compounds [9]. It consists of anionic polysaccharides and monosaccharides (42% D-galactose, 36–37% ribose, 7–20% D-glucuronic acid, 7% xylose, and 2% D-mannose), which are secreted by peach trees in response to injury [13]. Unlike synthetic coatings, PPR is non-toxic, renewable, and cost-efficient, making it an attractive alternative for large-scale food preservation applications [14].

Several studies have demonstrated PPR as a potential natural preservative for fresh produce, offering antioxidant, antimicrobial, and film-forming properties. The phenolic acids and flavonoids in PPR provide strong antioxidant properties, reducing oxidative stress and enzymatic browning [15]. Additionally, small amounts of proteins and enzyme inhibitors in PPR help suppress PPO and POD activity, delaying degradation and post-harvest browning [16]. PPR also exhibits strong antimicrobial properties, inhibiting post-harvest pathogens such as *Botrytis cinerea* and *Penicillium expansum*, thereby reducing fruit decay rates [17]. Its highly viscous, hydrophilic nature enables effective adhesion to fruit surfaces, forming a protective layer that minimizes moisture loss and oxidative damage [18]. Furthermore, PPR-based coatings demonstrate strong mechanical properties, including tensile strength and flexibility, making them ideal for post-harvest fruit preservation [19]. Several studies have confirmed PPR is very effective in preserving qualities of various perishable fruits [15,20]. Zhang et al. [15] found that PPR significantly delayed peach ripening by suppressing ethylene production and reducing fruit softening. Similarly, Yin et al. [20] reported that PPR coatings (1–4%) reduced browning in litchi by inhibiting PPO and LOX activity, with higher concentrations providing superior results. However, despite these promising findings, research on PPR’s effectiveness in controlling post-harvest browning in longkong remains limited. With its biodegradable, film-forming and coating properties, PPR presents a sustainable alternative to synthetic coatings, offering a viable solution for improving post-harvest fruit quality and extending shelf life.

Therefore, this study aims to investigate the effectiveness of PPR coatings at varying concentrations in inhibiting enzymatic browning and maintaining the post-harvest quality of longkong during ambient storage. By evaluating PPR’s role in suppressing browning-related enzymes such as PPO, POD, and LOX, as well as its ability to enhance pericarp stability, this research seeks to provide a sustainable and economically viable solution for extending longkong’s shelf life, thereby improving its marketability for both domestic and international trade.

## 2. Materials and methods

### 2.1. Chemicals and reagents

The chemicals and reagents used in this study were of analytical grade and these include ABTS (2,2-azino-bis (3-ethylbenzothiazoline-6-sulfonic acid)), aluminum chloride (AlCl₃), ammonium tetrarhodanatodiammonchromate, catechol, dithiothreitol, DPPH (2,2-diphenyl-1-picrylhydrazyl), ferric sulfate (FeSO₄), Folin-Ciocalteu reagent, guaiacol, hydrochloric acid (HCl), hydrogen peroxide, phosphatidylcholine (lecithin), polyvinylpolypyrrolidone (PVPP), potassium phosphate, sodium acetate, sodium carbonate, sodium chloride, sodium linoleate, sodium phosphate, thiobarbituric acid (TBA), and trichloroacetic acid (TCA). The chemicals used in the study were obtained from LOBA Chemie Pvt Ltd and Sigma Aldrich.

### 2.2. Raw material preparation, treatments and storage

**Fig 1:**
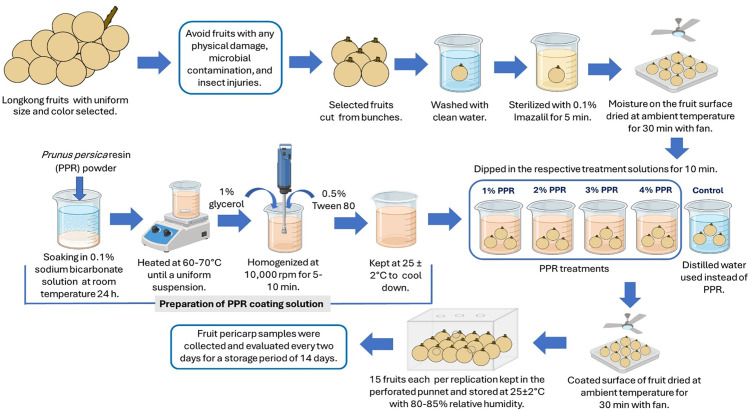
Infographic representation of PPR coating on the pericarp surface of longkong fruit and stored under ambient conditions.

Longkong fruits were harvested from a commercial orchard in the Nasan district, southern Thailand and transported to the laboratory on the same day. Fruits were inspected for physical damage, microbial contamination, and insect injuries, and those of uniform size and color were selected. The selected fruits were cut from the raceme end with the peduncle attached, washed thoroughly with clean water, and sterilized in 0.1% Imazalil solution for 5 min. After sterilization, they were surface air-dried using an electric fan to remove excess moisture. Then, fruits were divided into five groups: four treatment groups coated with PPR at 1%, 2%, 3%, and 4% (w/v) and one control group. The control group followed the same coating procedure but used distilled water instead of PPR. To prepare the PPR coating, the resin was first softened by soaking it in a 0.1% sodium bicarbonate solution for 24 h at room temperature to enhance its solubility. It was then heated in a water bath at 60–70°C under continuous stirring to facilitate dissolution. Subsequently, 1% glycerol was added as a plasticizer, along with 0.5% Tween 80 to stabilize the suspension. The mixture was then homogenized at 10,000 x g for 5–10 min using a handheld homogenizer (HR-6B, Beijing HiYi Technology Co., Ltd, China) to achieve a uniform dispersion. Following homogenization, the solution was gradually cooled to 25 ± 2°C, with occasional gentle stirring to prevent phase separation. Fruits in the treatment groups were immersed individually in their respective PPR coating emulsion for 10 min to ensure uniform coating. After immersion, fruits were air-dried under ambient conditions using an electric fan. The control group underwent the same dipping and drying process, but using distilled water instead of the PPR emulsion. All treated fruits were packaged in the punnet boxes (15 fruits per box) and stored at 25 ± 2°C with 80–85% relative humidity. Pericarp samples were collected every two days over a 14-day storage period to assess physicochemical and biochemical quality characteristics. Fig 1 illustrates the PPR coating procedure and its application to longkong fruits.

### 2.3. Quality analysis

#### 2.3.1. Color characteristics.

The pericarp color characteristics of longkong were evaluated according to the methodology of Venkatachalam et al. [3]. Color attributes, including lightness (L*), redness (a*), and yellowness (b*), were measured at 3–4 different points on the surface of 15 individual fruits for each treatment using a colorimeter (Hunter Lab, Reston, VA, USA).

#### 2.3.2. Browning Index (BI) and decay rate (DR).

The pericarp BI and DR were evaluated visually according to the methodology described by Liu et al. [21]. The scale for BI was as follows: 0 = no browning, 1 = less than 1/4 browning, 2 = 1/4–1/3 browning, 3 = 1/3–1/2 browning, 4 = 1/2–3/4 browning, and 5 = more than 3/4 browning. For DR, the scale was 0 = no visible fungal presence, 1 = less than 5% of surface affected, 2 = 5–20%, 3 = 20–50%, and 4 = more than 50%. Both BI and DR were calculated using the following equations:


$$\text{BI}=\;\;\;\;\;\;\;\sum \left(\frac{\text{browning }\;\;\;\;\;\text{ scale }\;\;\;\;\;\text{ x }\;\;\;\;\;\text{ number }\;\;\;\;\;\text{ of }\;\;\;\;\;\text{ fruit }\;\;\;\;\;\text{ in }\;\;\;\;\;\text{ each }\;\;\;\;\;\text{ class}}{5\;\;\;\;\;\;\text{ x }\;\;\;\;\;\text{ total }\;\;\;\;\;\text{ number }\;\;\;\;\;\text{ of }\;\;\;\;\;\text{ fruit }\;\;\;\;\;\text{ in }\;\;\;\;\;\text{ each }\;\;\;\;\;\text{ class}}\right)$$

$$\text{DR}=\;\;\;\;\;\;\;\sum \left(\frac{\text{decay }\;\;\;\;\;\text{ scale }\;\;\;\;\;\text{ x }\;\;\;\;\;\text{ fruit }\;\;\;\;\;\text{ within }\;\;\;\;\;\text{ each }\;\;\;\;\;\text{ class}}{\text{total }\;\;\;\;\;\text{ fruit }\;\;\;\;\;\text{ x }\;\;\;\;\;\text{ the }\;\;\;\;\;\text{ highest }\;\;\;\;\;\text{ score}}\right)\text{x }\;\;\;\;\;\;100$$

#### 2.3.3. Pericarp thickness and moisture loss.

Pericarp thickness was measured at 3–4 different points per treatment using a digital vernier caliper for each replicate. Results were reported in millimeters (mm).

Moisture loss in longkong pericarp was assessed following the method of Liu et al. [21]. It was calculated by comparing the initial moisture content at the start of storage with that at subsequent sampling intervals. Results were expressed as percentage (%).

#### 2.3.5. Electrical conductivity (EC).

The EC of longkong pericarp was measured using a modified method based on Wang et al. [22]. Twenty pericarp discs per replication were excised using a metal borer, rinsed with distilled water to remove surface electrolytes, and gently air-dried on absorbent paper. Each disc was placed in a test tube containing 20 mL of distilled water and incubated in a water bath at 25°C for 30 min to facilitate ion leakage. The initial electrical conductivity (L₀, S m^-1^) was recorded using a conductivity meter. The tubes were then heated to 100°C for 20 min to release all residual electrolytes, rapidly cooled to room temperature, and the final electrical conductivity (L₁, S m^-1^) was measured.

The relative electrical conductivity (EC) was calculated using the following formula:


$$\text{Relative }\;\;\;\;\;\text{ EC }\;\;\;\;\;\;\left(\text{S }\;\;\;\;\;\;m^{-1}\right)\;\;\;\;\;\;\;=\left(\frac{{\text{L}}_{0}}{{\text{L}}_{1}}\right)\;\;\;\;\;\;\;$$

#### 2.3.6. Malondialdehyde (MDA) content.

MDA content in longkong pericarp was measured by following the method of Noonim and Venkatachalam [6]. Pericarp (2 g) was homogenized in 20 mL of 50 mM sodium phosphate buffer (pH 7.8) and centrifuged at 12,000 × g for 15 min at 4°C. 2 mL of the supernatant was mixed with 3 mL of thiobarbituric acid (TBA) solution (5 g/L) in 0.1% trichloroacetic acid (TCA). The mixture was heated in a boiling water bath for 15 min, rapidly cooled, and centrifuged under the same conditions. Absorbance of the supernatant was measured at 450, 532, and 600 nm, and MDA content was calculated and expressed as nmol g^-1^.

#### 2.3.7. Phytochemical analysis.

For phytochemical analysis, 0.1 g of longkong pericarp was mixed with 2 mL of methanol and water solution (60/40, v/v), vortexed for 2 min, incubated at 60°C for 30 min, and then centrifuged at 4000 x g for 10 min. After that, the supernatant was collected and analyzed of the followings:

Total phenolic content (TPC) was determined using the Folin-Ciocalteu (FC) method, as described by Noonim and Venkatachalam [6]. A 0.1 mL aliquot of the supernatant was mixed with 0.6 mL of FC reagent, followed by a 2 min reaction period. Then, 0.48 mL of 7.5% sodium carbonate solution was added, and the mixture was vortexed for 1 min, followed by incubation for 30 min in the dark at room temperature. The absorbance was measured at 760 nm using a UV-Vis spectrophotometer (Mini UV 1240, Shimadzu, Kyoto, Japan). Gallic acid was used as a reference standard. TPC was expressed as mg gallic acid equivalents per gram of sample (mg GAE g^-1^).

Total flavonoid content (TFC) was determined by following the method of Yin et al. [20] with some modifications. A 0.5 mL aliquot of the extract was mixed with 0.5 mL of 2% aluminum chloride (AlCl₃) solution. Then, the reaction mixture was incubated at room temperature for an hour and then measured of absorbance at 420 nm using a UV-Vis spectrophotometer (Mini UV 1240, Shimadzu, Kyoto, Japan). Quercetin was used as a reference standard. TFC was expressed as μg quercetin equivalents (μg QE) per gram of sample (μg QE g^-1^).

#### 2.3.8. Antioxidant activities.

For antioxidant activities, 5 g of longkong pericarp samples were homogenized under cold conditions with 20 mL of 0.9% sodium chloride solution for 5 min. The homogenate was then centrifuged at 12,000 × g for 10 min in refrigerated conditions. The resulting supernatant was collected and used for the following antioxidant activity assays:

DPPH radical scavenging activity was determined by following the method of Brand-Williams et al. [23]. A 100 µL aliquot was thoroughly mixed with 3.9 mL of 60 µmol/L DPPH solution in a test tube. The mixture was incubated for 30 min in the dark at room temperature. Absorbance was measured at 515 nm using a spectrophotometer (Mini UV 1240, Shimadzu, Kyoto, Japan), and the results were expressed as %.

ABTS radical scavenging activity was determined by following the method of Lo’ay et al. [24]. A 0.1 mL aliquot was combined with 3.9 mL of ABTS reagent and incubated for 6 min at an ambient temperature. The absorbance was measured at 734 nm using a UV-Vis spectrophotometer (Mini UV 1240, Shimadzu, Kyoto, Japan), and the results were expressed as %.

#### 2.3.9. Enzyme Activities.

Browning-related enzyme activities in longkong pericarp were measured by following the method of Zhang et al. [25]. Pericarp sample (2g) was homogenized with 0.10 g of PVPP in 10 mL of 0.1 M phosphate buffer (pH 6.8). Then, the homogenate was centrifuged at 10,000 × g for 15 min under refrigerated conditions, and the supernatant was collected for PPO and POD activity assays. For PPO activity, 0.5 mL of supernatant was mixed with 2 mL of phosphate buffer, followed by 0.5 mL of 20 mM catechol solution. The reaction mixture absorbance was measured at 420 nm using a spectrophotometer (Mini UV 1240, Shimadzu, Kyoto, Japan). One unit of enzyme activity was defined as a 0.01 absorbance change per min, and expressed as units per gram (U g^-1^). For POD activity, 0.2 mL of enzyme extract was mixed with 2.8 mL of reaction mixture containing 50 mM phosphate buffer (pH 6.0), 20 mM guaiacol, and 25 mM hydrogen perioxide. The increase in absorbance at 470 nm was recorded using a spectrophotometer (Mini UV 1240, Shimadzu, Kyoto, Japan). Enzyme activity was expressed as U g^-1^

C4H activity in longkong pericarp was determined by following the method of Guo et al. [18]. A 0.5 g pericarp sample was homogenized in 5 mL of 200 mM phosphate buffer (pH 7.5) and centrifuged at 10,000 × g for 15 min under refrigerated conditions. For the reaction, 1 mL of supernatant was mixed with 1 mL of phosphate buffer containing 50 mM phosphate, 2 mM trans-cinnamic acid, 0.5 mM NADP-Na, and 2 mM mercaptoethanol. The mixture was incubated at 37°C for 1 h, and the reaction was stopped by adding 50 μL of 6 M hydrochloric acid. Absorbance was measured at 290 nm using a spectrophotometer (Mini UV 1240, Shimadzu, Kyoto, Japan). Enzyme activity was expressed as U g^-1^.

LOX activity in longkong pericarp was measured by following the method of Chareonphun et al. [7]. Pericarp sample (5 g) was homogenized in 10 mL of 50 mM potassium phosphate buffer (pH 7.0) under cold conditions (4°C). The homogenate was centrifuged at 15,000 × g for 15 min at 4°C, and the supernatant was collected for LOX activity analysis. The reaction mixture was prepared by mixing 0.2 mL of crude enzyme extract with 2.8 mL of potassium phosphate buffer containing 25 mM sodium linoleate as the substrate. Absorbance was measured at 234 nm using a UV-Visible spectrophotometer. LOX activity was defined as the enzyme quantity required to induce a 0.01 absorbance change per minute and expressed as U g^-1^.

PLD activity in longkong pericarp was measured by following the method of Chareonphun et al. [7], with some modifications. Pericarp samples (5 g) were pulverized in liquid nitrogen using a mortar and pestle and then homogenized with 25 mL of 0.1 M Tris-HCl buffer (pH 7.0). The mixture was centrifuged at 13,000 × g for 30 min at 4°C, and the supernatant was collected for PLD activity analysis. The reaction substrate was prepared by dissolving 0.05 g of phosphatidylcholine in 3 mL each of chloroform and purified water. The solvent was evaporated at 40°C using a rotary evaporator, and the dried substrate was rehydrated in 250 mL of acetate buffer (100 mM, pH 5.5) supplemented with 5 mM dithiothreitol and 25 mM calcium chloride. To initiate the reaction, 1 mL of crude enzyme extract was mixed with 3 mL of rehydrated substrate and agitated at room temperature for 60 min. Petroleum ether was then added for phase separation, followed by the introduction of an ammonium tetrarhodanatodiammonchromate solution (2 g in 100 mL methanol) to the aqueous phase, leading to precipitate formation. The precipitate was isolated via centrifugation (26,000 × g for 15 min), pellets were collected and dissolved in 3 mL of acetone, and analyzed using a UV-Vis spectrophotometer (Mini UV 1240, Shimadzu, Kyoto, Japan) at 520 nm. PLD activity was expressed as U g^-1^, where one unit corresponds to the generation of 1 µmol of choline per minute under assay conditions.

### 2.4. Statistical analysis

All experiments were conducted in triplicate, and results are presented as mean ± standard deviation. A completely randomized design was used. Data were analyzed using repeated measures ANOVA to assess the effects of treatment, storage time, and their interaction. Tukey’s HSD post-hoc test was applied to determine significant differences between treatment groups (P < 0.05). All statistical analyses were performed using SPSS software (version 12.0 for Microsoft Windows).

## 3. Results and discussion

### 3.1. Color characteristics

**Fig 2:**
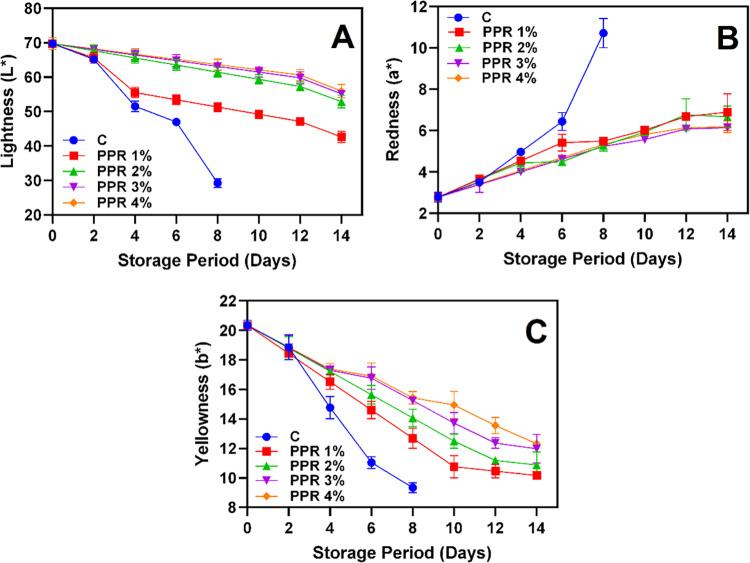
Lightness **(A)**, redness **(B)** and yellowness **(C)** of the pericarp of longkong fruit that coated with or without PPR coating and stored under ambient conditions (n = 3). Note: C represents the control group (uncoated); PPR 1%, PPR 2%, PPR 3%, and PPR 4% represent fruits coated with PPR solutions at 1%, 2%, 3%, and 4% concentrations, respectively.

Edible polymer coatings effectively control the pericarp color loss by creating a modified atmosphere that reduces respiration and delays senescence [1]. The color changes in longkong pericarp coated with varying concentrations of PPR and stored under ambient conditions are shown in Fig 2A–C. Longkong pericarp is highly sensitive to browning, which accelerates within a few days of storage due to the activation of browning-related enzymes [6]. This study observed a similar trend, with L* values decreasing significantly across all samples (Fig 2A). The control group exhibited the fastest decline, reaching L* values lower as 29.26 by day 8, after which severe browning and decay rendered measurements unfeasible. In contrast, PPR 3% and PPR 4% treatments slowed the decline, maintaining higher L* values throughout storage, indicating effective browning inhibition. Conversely, a* values increased gradually in all samples, signaling progressive browning (Fig 2B). The control group showed the steepest rise, reaching an a* value of 10.91 on day 8. Among the treatments, PPR 4% treated samples exhibited the lowest a* increase, followed by PPR 3%, suggesting that higher PPR concentrations effectively suppressed enzymatic browning. Similarly, b* values declined in all samples, with the control group experiencing the most rapid drop (from 20.34 at day 0 to 9.34 by day 8) (Fig 2C). However, PPR 4% treated samples were retained higher b* values, preserving yellow pigments essential for the fruit’s visual appeal. These changes in L*, a*, and b* values align with typical browning processes in longkong, primarily driven by phenolic oxidation catalyzed by PPO and POD enzymes [1,25]. However, the PPR coated samples had effectively mitigated these color changes by regulating browning-related enzyme activities (see Fig 7). Tahir et al. [26] found that a natural resin coating of gum Arabic inhibited PPO and POD activities in blueberries by forming a physical barrier, scavenging free radicals, and protecting enzyme substrates, effectively preventing oxidative browning and preserving fruit quality. The control samples exhibited a sharp decline in L* and b* values, along with a rapid increase in a* values, reflecting accelerated browning due to the absence of protective measures. PPR coatings, particularly at concentrations ≥ 3%, significantly delayed pericarp browning by forming a physical barrier that limits oxygen availability, thereby reducing enzymatic oxidation. This is in accordance with Yang Yong-li et al. [27], who demonstrated that a natural resin coating applied at concentrations above 3% significantly delayed pericarp browning in Chinese bayberry. Additionally, PPR coatings enhanced fruit skin integrity, minimized damage during handling, and provided preservative effects, collectively improving color retention and overall fruit quality during storage [28–30].

**Fig 3:**
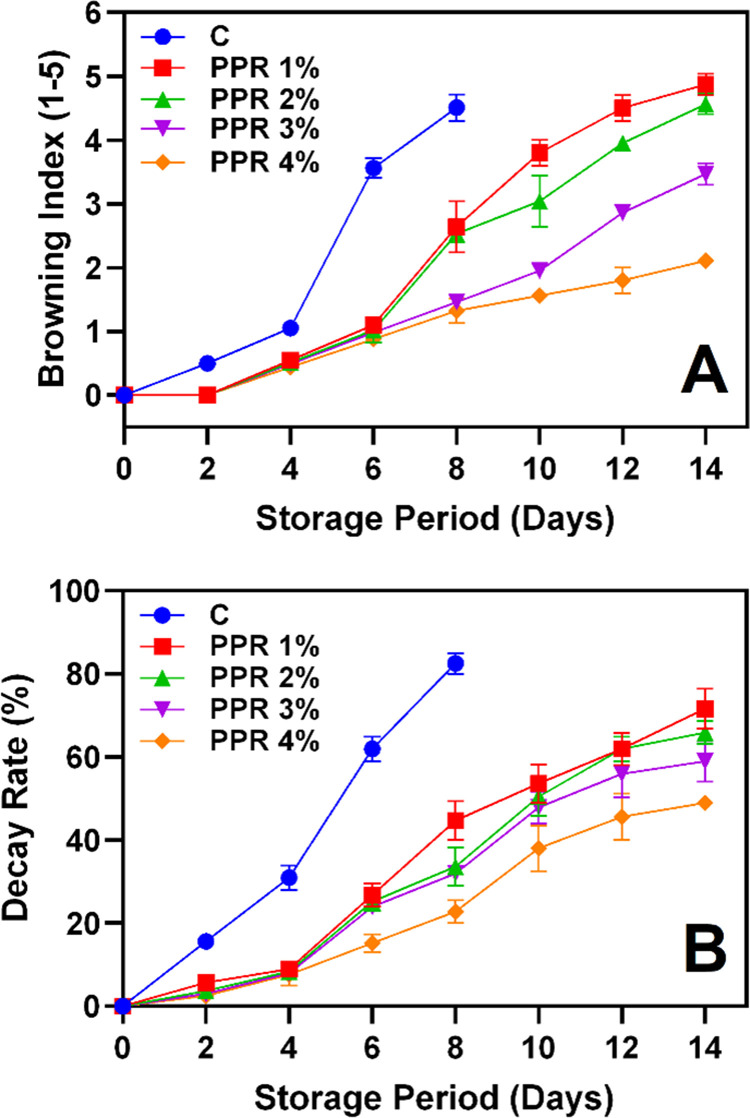
Browning index **(A)** and decay rate **(B)** of the pericarp of longkong fruit that coated with or without PPR coating and stored under ambient conditions (n = 3). Note: C represents the control group (uncoated); PPR 1%, PPR 2%, PPR 3%, and PPR 4% represent fruits coated with PPR solutions at 1%, 2%, 3%, and 4% concentrations, respectively.

**Fig 4:**
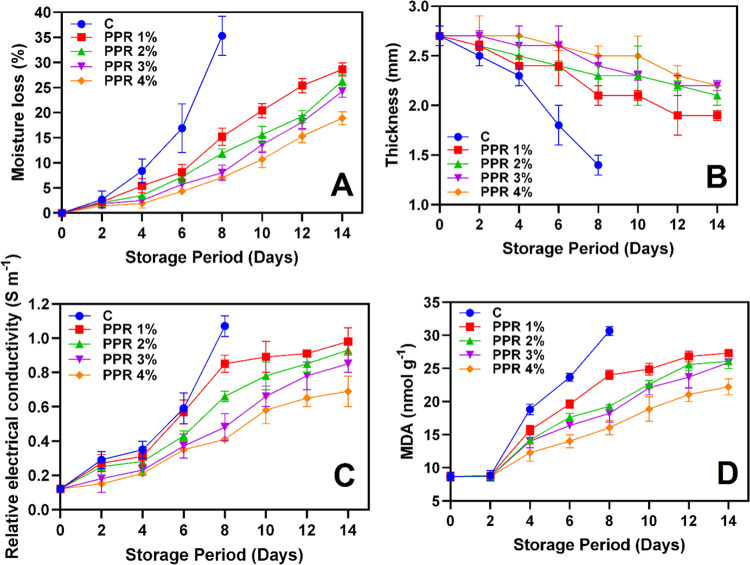
Moisture loss **(A)**, thickness **(B)**, electrical conductivity **(C)** and malondialdehyde content **(D)** of the pericarp of longkong fruit that coated with or without PPR coating and stored under ambient conditions (n = 3). Note: C represents the control group (uncoated); PPR 1%, PPR 2%, PPR 3%, and PPR 4% represent fruits coated with PPR solution at 1%, 2%, 3%, and 4% concentrations, respectively.

**Fig 5:**
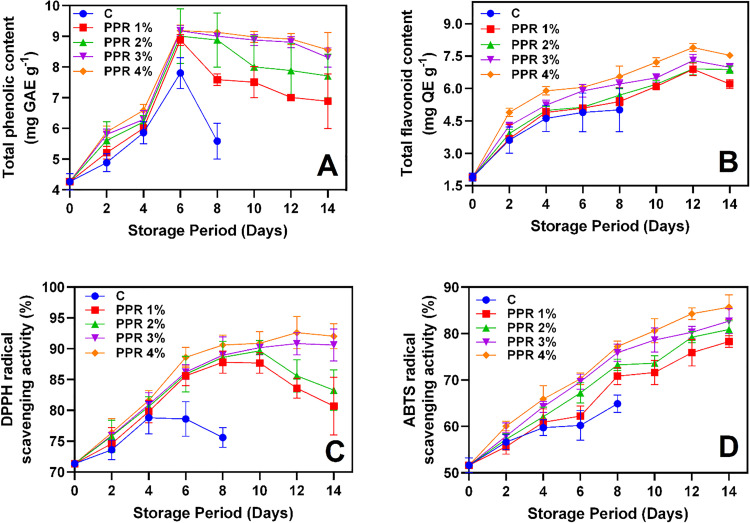
Total phenolic content **(A)**, total flavonoid content **(B)**, DPPH **(C)** and ABTS **(D)** radial scavenging activities of the pericarp of longkong fruit that coated with or without PPR coating and stored under ambient conditions (n = 3). Note: C represents the control group (uncoated); PPR 1%, PPR 2%, PPR 3%, and PPR 4% represent fruits coated with PPR solutions at 1%, 2%, 3%, and 4% concentrations, respectively.

**Fig 6:**
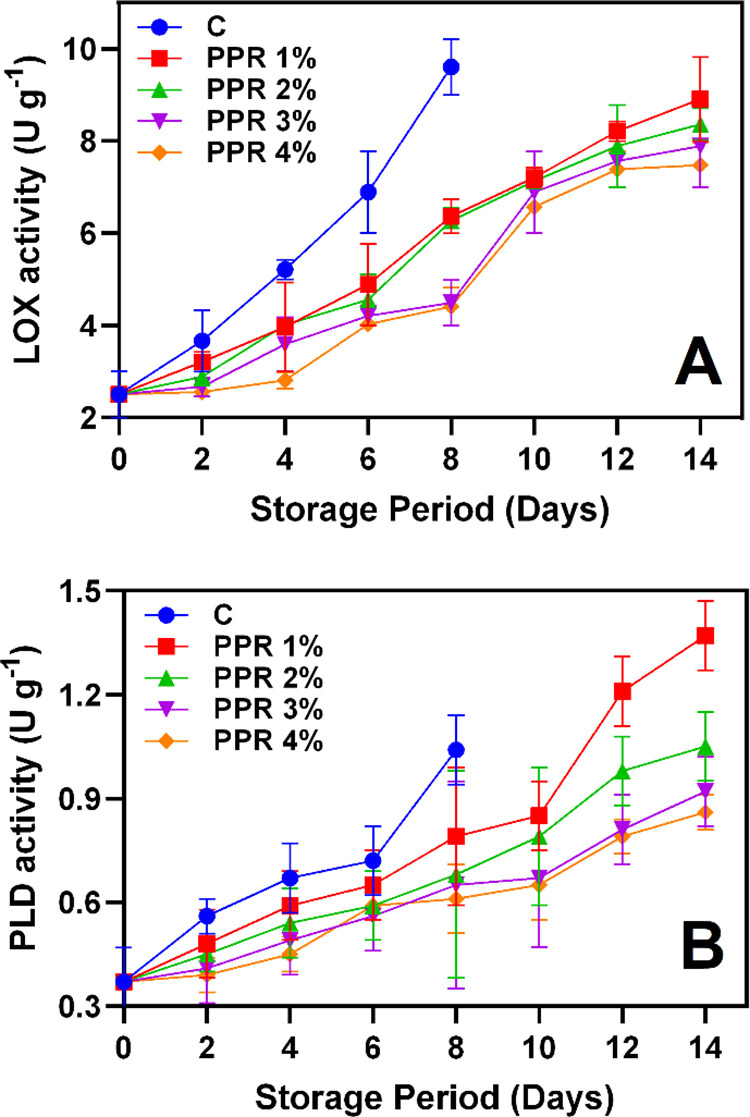
LOX **(A)** and PLD **(B)** activities of the pericarp of longkong fruit that coated with or without PPR coating and stored under ambient conditions (n = 3). Note: C represents the control group (uncoated); PPR 1%, PPR 2%, PPR 3%, and PPR 4% represent fruits coated with PPR solutions at 1%, 2%, 3%, and 4% concentrations, respectively.

**Fig. 7:**
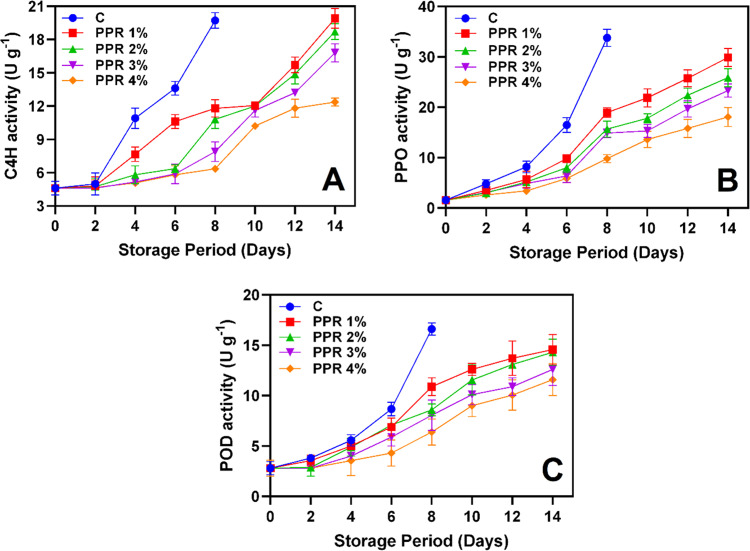
C4H (A), PPO (B) and POD (C) activities of the pericarp of longkong fruit that coated with or without PPR coating and stored under ambient conditions (n=3). Note: C represents the control group (uncoated); PPR 1%, PPR 2%, PPR 3%, and PPR 4% represent fruits coated with PPR solutions at 1%, 2%, 3%, and 4% concentrations, respectively.

### 3.2. Browning index and decay rate

The progressive increase in BI and DR during storage is a distinguishing trait of longkong fruit, which is highly prone to postharvest quality deterioration caused by enzymatic browning and microbial activity [3]. The BI serves as a key indicator of pericarp browning, reflecting the oxidation of polyphenols, membrane lipid degradation, and alterations in enzyme activity [31]. BI values increased progressively in all fruit samples during storage (Fig 3A). The control group exhibited the highest BI, exceeding 4.51 by day 8, indicating severe browning. In contrast, PPR-coated fruits had significantly lower BI values throughout storage. Among the treatments, PPR 3% and PPR 4% were the most effective, with final BI values of 3.47 and 2.11, respectively, by day 14, demonstrating the protective role of PPR coatings, particularly at higher concentrations. Meighani et al. [32] found that natural gum coatings on pomegranate effectively controlled respiration rate and water loss, thereby reducing browning intensity. Similarly, Sarpong et al. [33] reported that BI elevation in fruit pericarp is primarily due to cellular disintegration and the release of reactive species, including reducing sugars, amino acids, ascorbic acid, and phenolic compounds, which drive enzymatic and non-enzymatic browning. However, applying natural edible gums or resins forms a protective barrier, reducing oxygen exposure and slowing enzymatic oxidation [34]. Additionally, the antioxidant properties of PPR may contribute to ROS suppression, further delaying browning [35]. The increase in DR under ambient conditions results from physiological, biochemical, and enzymatic changes, which accelerate at higher temperatures and humidity levels [1,36]. DR progressively increased in all groups throughout storage (Fig 3B). By day 8, the control group reached a DR of 82.56%, indicating severe decay, after which it was excluded from the study due to being unmeasurable. PPR-treated fruits exhibited a significantly lower DR, with PPR 4% showing the least decay (48.99%), followed by PPR 3% (58.99%) on day 14, demonstrating the coating’s efficacy in slowing decay and preserving fruit quality. The lower DR in PPR-coated fruits suggests that the coating acted as a microbial barrier, reducing moisture loss and preventing fungal infections. Chen et al. [12] reported that PPR polysaccharides exhibit strong antibacterial activity, while Liu et al. [37] found that polyphenols, particularly flavonoids in PPR, enhance fungal resistance, contributing to its preservative effect on longkong fruit.

### 3.3. Moisture loss, thickness, EC and MDA content

Moisture loss in fruits primarily occurs through transpiration and diffusion via the cuticle and stomata [38]. Additionally, stress conditions can activate enzymatic processes in fruits, leading to cell membrane degradation, weakened cell walls, and reduced turgor pressure, which further accelerate moisture loss [39]. The present study showed a steady increase in moisture loss in all samples during all over the storage (Fig 4A). The control group exhibited the highest moisture loss, reaching 35.31% by day 8, indicating significant desiccation. In contrast, PPR-coated fruits retained more moisture, with PPR 4% showing the lowest moisture loss (<20%) throughout storage. Edible gum coatings form a thin protective film, acting as a barrier against moisture loss by reducing water vapor transmission between the pericarp and the surrounding environment [40,41]. Lin et al. [42] reported a strong correlation between water loss and pericarp thinning during storage, indicating that pericarp desiccation also affected pericarp thickness. The control group exhibited the fastest decline in pericarp thickness, reducing from 2.7 to 1.4 mm by day 8 (Fig 4B). In contrast, PPR-treated fruits, particularly PPR 4%, maintained greater thickness (2.2 mm by day 14), demonstrating the structural integrity provided by PPR coatings in minimizing shrinkage and maintaining fruit quality. Similarly, EC levels were increased progressively in all groups throughout the storage (Fig 4C). The raise of EC values in samples during storage is due to cell membrane degradation, loss of tissue integrity, and changes in ionic content [43]. The control group exhibited the highest EC values, exceeding 1.07 S m^-1^ by day 8, indicating significant membrane damage. PPR-treated fruits, particularly PPR 4%, showed the lowest EC values (0.69 S/m), demonstrating their role in preserving membrane integrity. Several studies suggest that controlled EC levels in natural resin- or gum-coated fruits correlate with lower MDA levels and enhanced antioxidant capacity [44–46]. MDA is a key marker of lipid peroxidation and oxidative stress in plants and this study found a continuous increase in longkong pericarp during storage (Fig 4D). The control group exhibited the highest MDA accumulation, peaking at 30.67 nmol g^-1^ by day 8, indicating severe oxidative damage. In contrast, PPR-treated fruits, particularly PPR 4%, exhibited significantly lower MDA levels, suggesting reduced oxidative stress and membrane deterioration. Overall, the higher EC and MDA levels in the control group reflect greater membrane damage and oxidative stress, which are key contributors to post-harvest quality loss. However, PPR coatings helped mitigate these effects, likely due to their antioxidant properties and barrier-forming capacity [37,47]. Huang et al. [48] found that Gum Arabic resin controlled MDA accumulation in Ponkan fruit by enhancing antioxidant enzymes, reducing lipid peroxidation, and forming an oxygen barrier to limit oxidative stress. This delayed senescence, preserved cellular integrity, and significantly lowered MDA levels, maintaining fruit quality during storage. Among the treatments, PPR 4% exhibited the most pronounced protective effects, making it the most effective concentration for preserving longkong fruit quality during ambient storage.

### 3.4. Phytochemicals and antioxidant activities

Phytochemicals such as TPC and TFC in longkong that treated with and without PPR coating are shown in Fig 5A–B. TPC levels increased across all groups during the initial storage period, peaking between days 2 and 4 (Fig 5A). After day 6, the control group exhibited a sharp decline, whereas PPR-treated fruits maintained higher TPC values throughout the 14-day storage period. Among the treatments, PPR 4% showed the highest TPC, reaching 9.13 mg GAE g^-1^ by day 8 and exhibiting minimal reduction (8.56 mg GAE g^-1^) by day 14. Similarly, TFC level also continuously increased in all samples (Fig 5B). The control group exhibited the lowest TFC, while PPR-treated fruits consistently outperformed the control. Among the treatments, PPR 4% maintained the highest TFC, nearing 7.53 mg QE g^-1^ by day 14. The increase in TPC and TFC during storage may result from the enzymatic degradation of cell wall components, which releases bound phenolics and flavonoids [8]. Lin et al. [42] reported that cell wall-degrading enzymes, including pectin esterase, polygalacturonase, and cellulase, break down structural polysaccharides such as protopectin, cellulose, and hemicellulose, thereby facilitating the release of phenolic compounds. The increase in polyphenolic compounds is linked to enzymatic activity in the phenylpropanoid biosynthetic pathway, where C4H (cinnamate 4-hydroxylase) plays a key role in flavonoid and lignin biosynthesis. This process enhances antioxidant activity and stress tolerance in plants [49]. This aligns with our findings, as C4H activity was elevated in PPR-treated samples. The sharp decline in TPC and TFC in the control groups is likely resulting from oxidative degradation caused by PPO and POD due to the lack of protective barriers. In contrast, PPR treatments effectively preserved phenolic and flavonoid compounds, likely by forming a barrier against oxygen and light exposure. Among the treatments, PPR 4% was the most effective in maintaining these phytochemicals. Meighani et al. [50] reported that a natural resin coating on pomegranate fruit effectively limited oxygen diffusion, reducing PPO and POD activities, slowing metabolic breakdown, and consequently retaining more bioactive compounds. DPPH radical scavenging activity steadily increased in all samples during storage (Fig 5C). However, after day 4, the control group exhibited reduced activity, while PPR-treated fruits maintained higher levels. PPR 4% showed the highest radical scavenging activity, sustaining 92.05% by day 14. Fruits treated with PPR 1%–2% gradually declined in DPPH radical scavenging activity after day 8, with this trend continuing until the end of storage. Similarly, ABTS radical scavenging activity increased across all samples throughout storage despite sample variations (Fig 5D). The control group showed the lowest activity, stabilizing at 64.89% by day 8, whereas PPR-treated fruits exhibited higher antioxidant activity, with PPR 4% reaching approximately 85.67% by day 14. Differences in DPPH and ABTS activity may be attributed to antioxidant stability and reactivity variations under different storage conditions. Valentina and Neelamegam [51] reported that factors such as temperature, light, and oxygen exposure affect antioxidant performance. The higher DPPH and ABTS radical scavenging activities in PPR-treated fruits suggest enhanced antioxidant preservation compared to the control. This preservation is likely due to PPR’s ability to reduce oxidative stress by limiting oxygen and reactive oxygen species (ROS) exposure. Among the treatments, PPR 4% consistently exhibited the strongest protective effects, reinforcing its potential as a natural preservative for extending the post-harvest shelf life of longkong fruit. This aligns with Carvalho et al. [52], who reported that applying a higher concentration of natural resin on sweet oranges created a stronger oxygen barrier, reducing oxidative stress and enzymatic degradation of antioxidants, thereby enhancing shelf life.

### 3.5. Enzyme activities

#### 3.5.1. Membrane degradative enzymes.

LOX is a key enzyme in lipid peroxidation, generating ROS in plants [53]. It contributes to the breakdown of cellular structures, accelerating senescence and browning in fruits [54]. In this study, LOX activity increased continuously in all groups throughout storage (Fig 6A). The control group exhibited the highest LOX activity, reaching 9.61 U g^-1^ by day 8, indicating substantial oxidative damage and membrane destabilization. In contrast, PPR-treated fruits showed significantly lower LOX activity, especially samples treated with PPR 4% demonstrating the greatest inhibition, maintaining activity below 7.48 U g^-1^ throughout storage. The antioxidant properties and polysaccharide content of PPR effectively suppress LOX activation, reducing oxidative stress and pericarp deterioration [55]. Generally, antioxidants neutralize ROS by stabilizing the cellular redox environment, disrupting oxidative stress signaling, and preventing membrane lipid peroxidation [56]. This aligns with our findings, as reduced LOX activity in PPR-treated samples correlated with improved longkong pericarp integrity and antioxidant activities. Similarly, PLD activity, which catalyzes the hydrolysis of membrane phospholipids [57], increased throughout storage (Fig 6B). The control group exhibited the highest PLD activity, peaking at 1.04 U g^-1^ on day 8, indicating extensive membrane degradation. In contrast, PPR 4% consistently exhibited the lowest PLD activity, remaining below 0.86 U g^-1^ throughout the storage period. The reduction in PLD activity in PPR-treated samples suggests that the PPR coating effectively stabilizes cellular membranes and mitigates enzymatic degradation. This may be attributed to PPR forming a protective barrier, reducing substrate availability for PLD activation and thereby inhibiting the production of phosphatidic acid, a key molecule in oxidative stress signaling and cell damage. Ali et al. [58] reported that edible gum coatings on ‘Kinnow’ mandarin significantly controlled LOX and PLD activity by forming a protective layer and maintaining membrane integrity. In contrast, the higher PLD activity in untreated samples aligns with their greater moisture loss, increased browning index, and higher decay rate, reinforcing that membrane destabilization is a key factor in pericarp quality deterioration.

#### 3.5.2. Browning related enzymes.

C4H influences pericarp color changes and the oxidative stress response in plants [59–60]. The present study found a continuous increase in C4H activity across all groups during storage (Fig 7A). The control group exhibited the highest activity, reaching 19.71 U g^-1^ by day 8, indicating increased polyphenolic synthesis and accelerated pericarp browning. In contrast, PPR-treated fruits, particularly those coated with PPR 4% exhibited the lowest C4H activity, which remained below 12.37 U g⁻¹ throughout the storage period. This suggests that PPR coatings effectively suppressed phenolic biosynthesis, thereby reducing enzymatic browning. C4H is typically activated in response to oxidative stress, which occurs when the balance between ROS production and the fruit’s ability to neutralize reactive intermediates is disrupted [61]. The lower C4H activity in PPR-treated fruits suggests reduced oxidative stress, likely due to the antioxidant properties of PPR. Natural plant resins contain bioactive compounds that enhance antioxidant activity, helping to mitigate oxidative stress [62]. PPO and POD are the key enzymes in enzymatic browning, significantly contributing to pericarp discoloration in longkong [1]. Both enzymes increased progressively in all samples, though PPR-treated fruits exhibited significantly lower levels (Fig 7B-C). PPO activity increased significantly (P < 0.05), with the control group peaking at 33.79 U g^-1^ by day 8. In contrast, PPR-treated fruits, particularly those treated with PPR 3% and PPR 4%, had lower PPO activity, remaining below 23 U g^-1^ throughout the storage period (Fig 7B). Similarly, POD activity increased steadily, with the control group reaching 16.61 Ug^-1^ by day 8, reflecting accelerated pericarp browning and oxidative damage (P < 0.05). However, PPR-treated fruits maintained significantly lower POD activity, with PPR 4% remaining below 12 U g^-1^ throughout storage (Fig 7C). PPO directly oxidizes phenolic compounds into quinones, leading to pericarp browning. On the other hand, POD requires hydrogen peroxide to oxidize phenolics, which explains the higher PPO activity in longkong compared to POD [63]. The reduced PPO and POD activity in PPR-treated fruits suggests that the coating effectively delayed enzymatic browning by forming a protective barrier that limits oxygen exposure and reduces substrate availability. Furthermore, the lower LOX and PLD activity in PPR-treated fruits indicates reduced cell membrane disintegration, preventing the release of enzymes and substrates contributing to pericarp browning. These findings align with previous studies demonstrating the effectiveness of natural coatings in reducing browning-related enzymatic activities in fruits [64–65]. The superior performance of PPR 4% compared to other treatments highlights a dose-dependent effect, reinforcing its efficacy in minimizing enzyme-induced quality degradation and extending post-harvest fruit quality.

## 4. Conclusion

This study demonstrated the effectiveness of PPR coatings in extending the shelf life of longkong fruit during ambient storage. Among the tested concentrations (PPR 1%, 2%, 3%, and 4%), PPR 4% was the most effective in preserving fruit quality. Untreated fruits deteriorated significantly within 8 days, exhibiting a higher browning index and an unacceptable decay rate. In contrast, fruits treated with PPR 4% had maintained acceptable quality throughout the storage period, with a browning index of 2.11 and a decay rate of 48.99%. The PPR 4% coating effectively delayed color degradation, reduced moisture loss, and preserved pericarp thickness. Additionally, it suppressed browning-related and membrane-degrading enzymes while maintaining higher levels of phenolic compounds, flavonoids, and antioxidant activities. These findings highlight that PPR 4% is the optimal concentration for post-harvest preservation of longkong, offering a natural, biodegradable alternative to synthetic preservatives. The application of PPR coatings not only extends the shelf life of longkong and possibly enhances its marketability, particularly benefiting small-scale farmers in tropical regions. Future research should explore the scalability of PPR coatings for commercial use, their efficacy on other perishable fruits, and the potential for integrating PPR with other natural preservation methods to further enhance post-harvest quality. This study underscores the potential of PPR coatings to reduce food waste, support sustainable agricultural practices, and improve economic outcomes for fruit producers.

## Supporting information

Data Availability This file contains the Data Availability. (DOCX)
