# Correction: Prevention and inhibition of post-harvest browning in longkong pericarp using *Prunus Persica* resin coating during ambient storage

**DOI:** 10.1371/journal.pone.0335761

**Published:** 2025-10-29

**Authors:** Narin Charoenphun, Somwang Lekjing, Karthikeyan Venkatachalam

There are errors in the author affiliations. The correct affiliations are as follows:

Narin Charoenphun ^1^, Somwang Lekjing ^2^, Karthikeyan Venkatachalam ^2^

1 Faculty of Science and Arts, Burapha University, Chanthaburi Campus, Chanthaburi, Thailand, 2 Faculty of Innovative Agriculture, Fisheries and Food, Prince of Songkla University, Surat Thani Campus, Makham Tia, Mueang, Surat Thani, Thailand
