# Supplementary material for: Prevention and inhibition of post-harvest browning in longkong pericarp using Prunus Persica resin coating during ambient storage
Source: PLoS One. 2025 May 12;20(5):e0323416. doi: 10.1371/journal.pone.0323416 (PMC12068716; doi:10.1371/journal.pone.0323416)
Supplement: Data Availability — (DOCX) [file pone.0323416.s001.docx]

| Lightness (L*) |  |  |  |  |  |  |  |  |  |  |
| --- | --- | --- | --- | --- | --- | --- | --- | --- | --- | --- |
| Storage period | Treatments | | | | | | | | | |
|  | Control | SD | T1 | SD | T2 | SD | T3 | SD | T4 | SD |
| 0 | 69.73 | 0.73 | 69.73 | 0.73 | 69.73 | 0.73 | 69.73 | 0.73 | 69.73 | 1.73 |
| 2 | 65.18 | 1.18 | 65.62 | 1.62 | 67.66 | 1.66 | 68.07 | 1.07 | 68.21 | 1.21 |
| 4 | 51.54 | 1.54 | 55.52 | 1.52 | 65.59 | 1.59 | 66.41 | 1.41 | 66.69 | 1.69 |
| 6 | 46.99 | 0.99 | 53.41 | 1.41 | 63.51 | 1.51 | 64.76 | 1.76 | 65.17 | 1.17 |
| 8 | 29.26 | 1.26 | 51.31 | 1.31 | 61.44 | 1.44 | 63.1 | 1.1 | 63.65 | 1.65 |
| 10 |  |  | 49.21 | 1.21 | 59.36 | 1.35 | 61.45 | 1.45 | 62.13 | 1.13 |
| 12 |  |  | 47.15 | 1.1 | 57.29 | 1.29 | 59.79 | 1.79 | 60.61 | 1.61 |
| 14 |  |  | 42.61 | 1.61 | 52.88 | 1.88 | 55.14 | 1.14 | 55.97 | 1.97 |
|  |  |  |  |  |  |  |  |  |  |  |

| Redness (a*) |  |  |  |  |  |  |  |  |  |  |
| --- | --- | --- | --- | --- | --- | --- | --- | --- | --- | --- |
| Storage period | Treatments | | | | | | | | | |
|  | Control | SD | T1 | SD | T2 | SD | T3 | SD | T4 | SD |
| 0 | 2.78 | 0.23 | 2.78 | 0.23 | 2.78 | 0.23 | 2.78 | 0.23 | 2.78 | 0.23 |
| 2 | 3.51 | 0.15 | 3.66 | 0.16 | 3.64 | 0.14 | 3.39 | 0.39 | 3.42 | 0.14 |
| 4 | 4.97 | 0.17 | 4.54 | 0.15 | 4.44 | 0.44 | 4 | 0.04 | 4.06 | 0.16 |
| 6 | 6.44 | 0.44 | 5.41 | 0.41 | 4.49 | 0.19 | 4.61 | 0.16 | 4.69 | 0.19 |
| 8 | 10.71 | 0.71 | 5.49 | 0.19 | 5.27 | 0.27 | 5.23 | 0.13 | 5.33 | 0.13 |
| 10 |  |  | 6.02 | 0.2 | 5.93 | 0.19 | 5.56 | 0.19 | 5.81 | 0.18 |
| 12 |  |  | 6.68 | 0.18 | 6.77 | 0.77 | 6.08 | 0.08 | 6.14 | 0.14 |
| 14 |  |  | 6.89 | 0.89 | 6.66 | 0.53 | 6.14 | 0.14 | 6.19 | 0.29 |
|  |  |  |  |  |  |  |  |  |  |  |

| Yellowness (b*) |  |  |  |  |  |  |  |  |  |  |
| --- | --- | --- | --- | --- | --- | --- | --- | --- | --- | --- |
| Storage period | Treatments | | | | | | | | | |
|  | Control | SD | T1 | SD | T2 | SD | T3 | SD | T4 | SD |
| 0 | 20.34 | 0.34 | 20.34 | 0.34 | 20.34 | 0.34 | 20.34 | 0.34 | 20.34 | 0.34 |
| 2 | 18.84 | 0.84 | 18.43 | 0.43 | 18.78 | 0.78 | 18.82 | 0.82 | 18.86 | 0.86 |
| 4 | 14.76 | 0.76 | 16.51 | 0.51 | 17.21 | 0.21 | 17.29 | 0.29 | 17.38 | 0.38 |
| 6 | 11.04 | 0.4 | 14.6 | 0.6 | 15.63 | 0.63 | 16.76 | 0.76 | 16.9 | 0.9 |
| 8 | 9.34 | 0.34 | 12.68 | 0.68 | 14.06 | 0.6 | 15.24 | 0.24 | 15.42 | 0.42 |
| 10 |  |  | 10.76 | 0.76 | 12.48 | 0.48 | 13.71 | 0.71 | 14.94 | 0.94 |
| 12 |  |  | 10.46 | 0.465 | 11.18 | 0.18 | 12.36 | 0.36 | 13.55 | 0.55 |
| 14 |  |  | 10.17 | 0.17 | 10.88 | 0.88 | 11.97 | 0.97 | 12.31 | 0.1 |
|  |  |  |  |  |  |  |  |  |  |  |

| Browning index (1-5) | |  |  |  |  |  |  |  |  |  |
| --- | --- | --- | --- | --- | --- | --- | --- | --- | --- | --- |
| Storage period | Treatments | | | | | | | | | |
|  | Control | SD | T1 | SD | T2 | SD | T3 | SD | T4 | SD |
| 0 | 0 | 0 | 0 | 0 | 0 | 0 | 0 | 0 | 0 | 0 |
| 2 | 0.5 | 0.1 | 0 | 0 | 0 | 0 | 0 | 0 | 0 | 0 |
| 4 | 1.05 | 0.1 | 0.55 | 0.1 | 0.51 | 0.1 | 0.49 | 0.09 | 0.44 | 0.1 |
| 6 | 3.56 | 0.16 | 1.1 | 0.1 | 1.02 | 0.2 | 0.98 | 0.08 | 0.88 | 0.1 |
| 8 | 4.51 | 0.21 | 2.64 | 0.4 | 2.53 | 0.13 | 1.46 | 0.063 | 1.32 | 0.2 |
| 10 |  |  | 3.8 | 0.2 | 3.04 | 0.4 | 1.95 | 0.06 | 1.56 | 0.1 |
| 12 |  |  | 4.5 | 0.2 | 3.95 | 0.015 | 2.86 | 0.06 | 1.8 | 0.2 |
| 14 |  |  | 4.87 | 0.17 | 4.56 | 0.16 | 3.47 | 0.17 | 2.11 | 0.1 |
|  |  |  |  |  |  |  |  |  |  |  |

| Decay rate (%) |  |  |  |  |  |  |  |  |  |  |
| --- | --- | --- | --- | --- | --- | --- | --- | --- | --- | --- |
| Storage period | Treatments | | | | | | | | | |
|  | Control | SD | T1 | SD | T2 | SD | T3 | SD | T4 | SD |
| 0 | 0 | 0 | 0 | 0 | 0 | 0 | 0 | 0 | 0 | 0 |
| 2 | 15.61 | 1.61 | 5.71 | 0.71 | 3.71 | 0.71 | 3.06 | 0.16 | 2.5 | 0.54 |
| 4 | 30.96 | 2.96 | 8.94 | 0.94 | 8.41 | 1.4 | 7.98 | 0.98 | 7.61 | 2.61 |
| 6 | 61.92 | 2.92 | 26.82 | 2.8 | 25.23 | 2.3 | 23.94 | 0.94 | 15.22 | 2.1 |
| 8 | 82.56 | 2.56 | 44.7 | 4.7 | 33.64 | 4.6 | 31.92 | 1.92 | 22.83 | 2.83 |
| 10 |  |  | 53.64 | 4.6 | 50.46 | 4.6 | 47.88 | 3.8 | 38.05 | 5.5 |
| 12 |  |  | 61.98 | 3.9 | 61.98 | 2.9 | 55.99 | 5.8 | 45.66 | 5.6 |
| 14 |  |  | 71.59 | 4.9 | 65.89 | 2.8 | 58.99 | 4.9 | 48.99 | 1.9 |
|  |  |  |  |  |  |  |  |  |  |  |

| Thickness (mm) |  |  |  |  |  |  |  |  |  |  |
| --- | --- | --- | --- | --- | --- | --- | --- | --- | --- | --- |
| Storage period | Treatments | | | | | | | | | |
|  | Control | SD | T1 | SD | T2 | SD | T3 | SD | T4 | SD |
| 0 | 2.7 | 0.1 | 2.7 | 0.1 | 2.7 | 0.1 | 2.7 | 0.1 | 2.7 | 0.1 |
| 2 | 2.5 | 0.1 | 2.6 | 0.05 | 2.6 | 0.05 | 2.7 | 0.05 | 2.7 | 0.2 |
| 4 | 2.3 | 0.1 | 2.4 | 0.1 | 2.5 | 0.2 | 2.6 | 0.2 | 2.7 | 0.1 |
| 6 | 1.8 | 0.2 | 2.4 | 0.2 | 2.4 | 0.05 | 2.6 | 0.2 | 2.6 | 0.05 |
| 8 | 1.4 | 0.1 | 2.1 | 0.1 | 2.3 | 0.1 | 2.4 | 0.1 | 2.5 | 0.1 |
| 10 |  |  | 2.1 | 0.05 | 2.3 | 0.3 | 2.3 | 0.05 | 2.5 | 0.2 |
| 12 |  |  | 1.9 | 0.2 | 2.2 | 0.05 | 2.2 | 0.1 | 2.3 | 0.1 |
| 14 |  |  | 1.9 | 0.05 | 2.1 | 0.1 | 2.2 | 0.05 | 2.2 | 0.05 |
|  |  |  |  |  |  |  |  |  |  |  |

| Moisture loss (%) |  |  |  |  |  |  |  |  |  |  |
| --- | --- | --- | --- | --- | --- | --- | --- | --- | --- | --- |
| Storage period | Treatments | | | | | | | | | |
|  | Control | SD | T1 | SD | T2 | SD | T3 | SD | T4 | SD |
| 0 | 0 | 0 | 0 | 0 | 0 | 0 | 0 | 0 | 0 | 0 |
| 2 | 2.67 | 1.67 | 2.21 | 0.67 | 2.05 | 0.5 | 1.85 | 0.85 | 1.41 | 0.41 |
| 4 | 8.37 | 2.37 | 5.41 | 1.4 | 3.49 | 0.49 | 2.49 | 0.49 | 1.89 | 0.9 |
| 6 | 16.89 | 4.89 | 8.15 | 1.5 | 7.21 | 1.2 | 5.71 | 0.71 | 4.31 | 0.31 |
| 8 | 35.31 | 3.9 | 15.17 | 1.7 | 11.89 | 0.89 | 8.01 | 1.5 | 7.05 | 0.51 |
| 10 |  |  | 20.41 | 1.4 | 15.61 | 1.6 | 13.61 | 1.6 | 10.69 | 1.6 |
| 12 |  |  | 25.39 | 1.4 | 19.24 | 1.2 | 18.13 | 1.3 | 15.31 | 1.3 |
| 14 |  |  | 28.63 | 1.3 | 26.32 | 1.3 | 24.24 | 1.2 | 18.91 | 1.3 |
|  |  |  |  |  |  |  |  |  |  |  |

| Electrical conductivity (S/m) | |  |  |  |  |  |  |  |  |  |
| --- | --- | --- | --- | --- | --- | --- | --- | --- | --- | --- |
| Storage period | Treatments | | | | | | | | | |
|  | Control | SD | T1 | SD | T2 | SD | T3 | SD | T4 | SD |
| 0 | 0.12 | 0.02 | 0.12 | 0.02 | 0.12 | 0.02 | 0.12 | 0.02 | 0.12 | 0.02 |
| 2 | 0.29 | 0.05 | 0.27 | 0.05 | 0.25 | 0.05 | 0.18 | 0.08 | 0.15 | 0.05 |
| 4 | 0.35 | 0.05 | 0.31 | 0.05 | 0.28 | 0.08 | 0.23 | 0.03 | 0.21 | 0.01 |
| 6 | 0.59 | 0.09 | 0.57 | 0.07 | 0.43 | 0.03 | 0.37 | 0.07 | 0.35 | 0.05 |
| 8 | 1.07 | 0.06 | 0.85 | 0.05 | 0.66 | 0.03 | 0.48 | 0.08 | 0.41 | 0.01 |
| 10 |  |  | 0.89 | 0.09 | 0.78 | 0.08 | 0.66 | 0.06 | 0.58 | 0.08 |
| 12 |  |  | 0.91 | 0.01 | 0.85 | 0.05 | 0.78 | 0.08 | 0.65 | 0.05 |
| 14 |  |  | 0.98 | 0.08 | 0.93 | 0.03 | 0.85 | 0.05 | 0.69 | 0.09 |
|  |  |  |  |  |  |  |  |  |  |  |

| MDA content (nmol/g ) | |  |  |  |  |  |  |  |  |  |
| --- | --- | --- | --- | --- | --- | --- | --- | --- | --- | --- |
| Storage period | Treatments | | | | | | | | | |
|  | Control | SD | T1 | SD | T2 | SD | T3 | SD | T4 | SD |
| 0 | 8.67 | 0.67 | 8.67 | 0.67 | 8.67 | 0.67 | 8.67 | 0.67 | 8.67 | 0.67 |
| 2 | 8.81 | 0.81 | 8.8 | 0.8 | 8.69 | 0.69 | 8.67 | 0.67 | 8.67 | 0.67 |
| 4 | 18.81 | 0.81 | 15.69 | 0.69 | 14.21 | 0.21 | 14.01 | 1.01 | 12.29 | 1.29 |
| 6 | 23.67 | 0.61 | 19.61 | 0.61 | 17.61 | 0.61 | 16.39 | 0.39 | 14.01 | 1.01 |
| 8 | 30.67 | 0.67 | 24.01 | 0.69 | 19.29 | 0.21 | 18.21 | 1.29 | 16.05 | 1.05 |
| 10 |  |  | 24.89 | 0.89 | 22.61 | 0.61 | 22.09 | 1.09 | 18.86 | 1.86 |
| 12 |  |  | 26.81 | 0.81 | 25.59 | 0.89 | 23.69 | 1.69 | 21.05 | 1.05 |
| 14 |  |  | 27.31 | 0.31 | 26.09 | 1.09 | 25.89 | 0.89 | 22.21 | 1.21 |
|  |  |  |  |  |  |  |  |  |  |  |

| Total phenolic level (mg GAE/g) | | | | | | | | | | |
| --- | --- | --- | --- | --- | --- | --- | --- | --- | --- | --- |
| Storage period | Treatments | | | | | | | | | |
|  | Control | SD | T1 | SD | T2 | SD | T3 | SD | T4 | SD |
| 0 | 4.27 | 0.27 | 4.27 | 0.27 | 4.27 | 0.27 | 4.27 | 0.27 | 4.27 | 0.27 |
| 2 | 4.89 | 0.29 | 5.21 | 0.21 | 5.61 | 0.61 | 5.81 | 0.18 | 5.89 | 0.19 |
| 4 | 5.86 | 0.36 | 6.01 | 0.1 | 6.21 | 0.21 | 6.29 | 0.29 | 6.59 | 0.19 |
| 6 | 7.81 | 0.51 | 8.88 | 0.18 | 9.01 | 0.89 | 9.18 | 0.18 | 9.19 | 0.19 |
| 8 | 5.59 | 0.59 | 7.59 | 0.19 | 8.88 | 0.88 | 9.01 | 0.1 | 9.13 | 0.1 |
| 10 |  |  | 7.51 | 0.51 | 8.01 | 1.01 | 8.88 | 0.18 | 8.98 | 0.18 |
| 12 |  |  | 7.01 | 0.1 | 7.89 | 0.89 | 8.81 | 0.18 | 8.91 | 0.18 |
| 14 |  |  | 6.89 | 0.89 | 7.71 | 0.71 | 8.31 | 0.31 | 8.56 | 0.56 |
|  |  |  |  |  |  |  |  |  |  |  |

| Total flavonoid level (µg QE/g ) | | |  |  |  |  |  |  |  |  |
| --- | --- | --- | --- | --- | --- | --- | --- | --- | --- | --- |
| Storage period | Treatments | | | | | | | | | |
|  | Control | SD | T1 | SD | T2 | SD | T3 | SD | T4 | SD |
| 0 | 1.89 | 0.19 | 1.89 | 0.19 | 1.89 | 0.19 | 1.89 | 0.19 | 1.89 | 0.19 |
| 2 | 3.61 | 0.61 | 3.67 | 0.67 | 3.91 | 0.19 | 4.27 | 0.17 | 4.89 | 0.19 |
| 4 | 4.61 | 0.61 | 4.89 | 0.19 | 4.98 | 0.187 | 5.23 | 0.23 | 5.89 | 0.19 |
| 6 | 4.89 | 0.89 | 5.09 | 0.1 | 5.13 | 0.13 | 5.89 | 0.29 | 6.05 | 0.1 |
| 8 | 5.01 | 1.01 | 5.39 | 0.19 | 5.69 | 0.29 | 6.21 | 0.21 | 6.55 | 0.5 |
| 10 |  |  | 6.09 | 0.1 | 6.19 | 0.19 | 6.49 | 0.19 | 7.21 | 0.21 |
| 12 |  |  | 6.89 | 0.29 | 6.91 | 0.29 | 7.29 | 0.29 | 7.89 | 0.19 |
| 14 |  |  | 6.21 | 0.21 | 6.87 | 0.17 | 6.98 | 0.18 | 7.53 | 0.15 |
|  |  |  |  |  |  |  |  |  |  |  |

| DPPH radical scavenging activity (%) | | |  |  |  |  |  |  |  |  |
| --- | --- | --- | --- | --- | --- | --- | --- | --- | --- | --- |
| Storage period | Treatments | | | | | | | | | |
|  | Control | SD | T1 | SD | T2 | SD | T3 | SD | T4 | SD |
| 0 | 71.37 | 0.5 | 71.37 | 0.5 | 71.37 | 0.5 | 71.37 | 0.5 | 71.37 | 0.5 |
| 2 | 73.61 | 1.6 | 74.61 | 2.6 | 75.81 | 2.6 | 75.94 | 2.5 | 76.39 | 2.3 |
| 4 | 78.81 | 2.61 | 79.81 | 1.81 | 80.61 | 1.61 | 80.93 | 1.93 | 81.61 | 1.61 |
| 6 | 78.61 | 2.81 | 85.61 | 1.61 | 85.89 | 2.89 | 86.21 | 1.21 | 88.61 | 1.61 |
| 8 | 75.61 | 1.61 | 87.81 | 1.81 | 88.61 | 2.61 | 88.96 | 2.96 | 90.61 | 1.61 |
| 10 |  |  | 87.69 | 1.68 | 89.65 | 1.65 | 90.19 | 1.19 | 90.89 | 1.89 |
| 12 |  |  | 83.61 | 1.61 | 85.61 | 2.61 | 90.85 | 1.85 | 92.61 | 2.61 |
| 14 |  |  | 80.69 | 4.69 | 83.29 | 3.29 | 90.61 | 2.61 | 92.05 | 2.05 |
|  |  |  |  |  |  |  |  |  |  |  |

| ABTS radical scavenging activity (%) | | |  |  |  |  |  |  |  |  |
| --- | --- | --- | --- | --- | --- | --- | --- | --- | --- | --- |
| Storage period | Treatments | | | | | | | | | |
|  | Control | SD | T1 | SD | T2 | SD | T3 | SD | T4 | SD |
| 0 | 51.63 | 1.63 | 51.63 | 1.63 | 51.63 | 1.63 | 51.63 | 1.63 | 51.63 | 1.63 |
| 2 | 56.61 | 1.61 | 55.61 | 1.61 | 57.21 | 1.21 | 57.89 | 2.89 | 60.05 | 1.05 |
| 4 | 59.71 | 1.71 | 60.89 | 2.89 | 62.09 | 2.09 | 64.21 | 1.21 | 65.89 | 2.89 |
| 6 | 60.21 | 3.21 | 62.21 | 2.21 | 67.21 | 2.21 | 69.61 | 1.61 | 70.25 | 1.25 |
| 8 | 64.89 | 1.89 | 70.81 | 1.81 | 73.25 | 1.25 | 75.89 | 1.89 | 77.21 | 1.21 |
| 10 |  |  | 71.61 | 2.61 | 73.61 | 1.61 | 78.61 | 2.61 | 80.61 | 2.61 |
| 12 |  |  | 75.89 | 2.89 | 79.21 | 1.21 | 80.27 | 1.27 | 84.29 | 1.29 |
| 14 |  |  | 78.29 | 1.29 | 80.89 | 1.89 | 82.67 | 2.67 | 85.67 | 2.67 |
|  |  |  |  |  |  |  |  |  |  |  |

| LOX activity (U/g) |  |  |  |  |  |  |  |  |  |  |
| --- | --- | --- | --- | --- | --- | --- | --- | --- | --- | --- |
| Storage period | Treatments | | | | | | | | | |
|  | Control | SD | T1 | SD | T2 | SD | T3 | SD | T4 | SD |
| 0 | 2.51 | 0.51 | 2.51 | 0.51 | 2.51 | 0.1 | 2.51 | 0.1 | 2.51 | 0.1 |
| 2 | 3.67 | 0.67 | 3.21 | 0.21 | 2.89 | 0.2 | 2.67 | 0.2 | 2.56 | 0.1 |
| 4 | 5.21 | 0.21 | 3.97 | 0.97 | 4.01 | 0.1 | 3.59 | 0.59 | 2.81 | 0.18 |
| 6 | 6.89 | 0.89 | 4.89 | 0.89 | 4.56 | 0.56 | 4.21 | 0.21 | 4.03 | 0.03 |
| 8 | 9.61 | 0.61 | 6.37 | 0.37 | 6.27 | 0.27 | 4.49 | 0.49 | 4.41 | 0.41 |
| 10 |  |  | 7.21 | 0.21 | 7.13 | 0.13 | 6.89 | 0.89 | 6.57 | 0.57 |
| 12 |  |  | 8.21 | 0.21 | 7.89 | 0.89 | 7.57 | 0.57 | 7.39 | 0.39 |
| 14 |  |  | 8.91 | 0.91 | 8.36 | 0.36 | 7.89 | 0.89 | 7.48 | 0.48 |
|  |  |  |  |  |  |  |  |  |  |  |

| PLD activity (U/g) |  |  |  |  |  |  |  |  |  |  |
| --- | --- | --- | --- | --- | --- | --- | --- | --- | --- | --- |
| Storage period | Treatments | | | | | | | | | |
|  | Control | SD | T1 | SD | T2 | SD | T3 | SD | T4 | SD |
| 0 | 0.37 | 0.1 | 0.37 | 0.1 | 0.37 | 0.1 | 0.37 | 0.1 | 0.37 | 0.1 |
| 2 | 0.56 | 0.05 | 0.48 | 0.1 | 0.45 | 0.05 | 0.41 | 0.1 | 0.39 | 0.05 |
| 4 | 0.67 | 0.1 | 0.59 | 0.1 | 0.54 | 0.1 | 0.49 | 0.1 | 0.45 | 0.05 |
| 6 | 0.72 | 0.1 | 0.65 | 0.1 | 0.59 | 0.1 | 0.56 | 0.1 | 0.59 | 0.1 |
| 8 | 1.04 | 0.1 | 0.79 | 0.2 | 0.68 | 0.3 | 0.65 | 0.3 | 0.61 | 0.1 |
| 10 |  |  | 0.85 | 0.1 | 0.79 | 0.2 | 0.67 | 0.2 | 0.65 | 0.1 |
| 12 |  |  | 1.21 | 0.1 | 0.98 | 0.1 | 0.81 | 0.1 | 0.79 | 0.05 |
| 14 |  |  | 1.37 | 0.1 | 1.05 | 0.1 | 0.92 | 0.1 | 0.86 | 0.05 |
|  |  |  |  |  |  |  |  |  |  |  |

| C4H activity (U/g) |  |  |  |  |  |  |  |  |  |  |
| --- | --- | --- | --- | --- | --- | --- | --- | --- | --- | --- |
| Storage period | Treatments | | | | | | | | | |
|  | Control | SD | T1 | SD | T2 | SD | T3 | SD | T4 | SD |
| 0 | 4.61 | 0.61 | 4.61 | 0.61 | 4.61 | 0.61 | 4.61 | 0.61 | 4.61 | 0.61 |
| 2 | 4.99 | 0.99 | 4.81 | 0.81 | 4.75 | 0.75 | 4.61 | 0.61 | 4.67 | 0.67 |
| 4 | 10.91 | 0.91 | 7.65 | 0.65 | 5.81 | 0.81 | 5.17 | 0.17 | 5.08 | 0.08 |
| 6 | 13.61 | 0.61 | 10.61 | 0.61 | 6.37 | 0.37 | 5.89 | 0.89 | 5.81 | 0.81 |
| 8 | 19.71 | 0.71 | 11.79 | 0.79 | 10.81 | 0.81 | 7.89 | 0.89 | 6.35 | 0.35 |
| 10 |  |  | 12.05 | 0.04 | 12.01 | 0.1 | 11.61 | 0.61 | 10.21 | 0.21 |
| 12 |  |  | 15.71 | 0.71 | 14.89 | 0.89 | 13.21 | 0.21 | 11.81 | 0.81 |
| 14 |  |  | 19.89 | 0.89 | 18.71 | 0.71 | 16.81 | 0.81 | 12.37 | 0.37 |
|  |  |  |  |  |  |  |  |  |  |  |

| PPO activity (U/g) |  |  |  |  |  |  |  |  |  |  |
| --- | --- | --- | --- | --- | --- | --- | --- | --- | --- | --- |
| Storage period | Treatments | | | | | | | | | |
|  | Control | SD | T1 | SD | T2 | SD | T3 | SD | T4 | SD |
| 0 | 1.57 | 0.7 | 1.57 | 0.1 | 1.57 | 0.1 | 1.57 | 0.1 | 1.57 | 0.1 |
| 2 | 4.83 | 0.83 | 3.51 | 0.51 | 3.01 | 0.1 | 3.11 | 0.1 | 2.61 | 0.61 |
| 4 | 8.19 | 1.19 | 5.67 | 1.6 | 5.17 | 0.17 | 4.89 | 0.89 | 3.41 | 0.5 |
| 6 | 16.47 | 1.5 | 9.81 | 0.81 | 8.01 | 1.01 | 6.37 | 1.3 | 5.89 | 0.89 |
| 8 | 33.79 | 1.7 | 18.96 | 0.96 | 15.67 | 1.6 | 14.89 | 0.89 | 9.81 | 0.81 |
| 10 |  |  | 21.89 | 1.8 | 17.81 | 0.91 | 15.31 | 1.3 | 13.61 | 1.6 |
| 12 |  |  | 25.76 | 1.7 | 22.39 | 1.4 | 19.67 | 1.6 | 15.81 | 1.8 |
| 14 |  |  | 29.89 | 1.8 | 25.89 | 1.8 | 23.33 | 1.3 | 18.09 | 1.9 |
|  |  |  |  |  |  |  |  |  |  |  |

| POD activity (U/g) | |  |  |  |  |  |  |  |  |  |
| --- | --- | --- | --- | --- | --- | --- | --- | --- | --- | --- |
| Storage period | Treatments | | | | | | | | | |
|  | Control | SD | T1 | SD | T2 | SD | T3 | SD | T4 | SD |
| 0 | 2.81 | 0.65 | 2.81 | 0.65 | 2.81 | 0.65 | 2.81 | 0.65 | 2.81 | 0.8 |
| 2 | 3.81 | 0.1 | 3.56 | 0.56 | 2.89 | 0.89 | 2.81 | 0.81 | 2.81 | 0.81 |
| 4 | 5.57 | 0.57 | 5.01 | 0.1 | 4.89 | 0.89 | 4.01 | 0.1 | 3.56 | 1.5 |
| 6 | 8.67 | 0.67 | 6.89 | 0.89 | 7.09 | 0.19 | 5.89 | 0.89 | 4.31 | 1.3 |
| 8 | 16.61 | 0.61 | 10.89 | 0.89 | 8.59 | 0.59 | 8.05 | 1.5 | 6.39 | 1.3 |
| 10 |  |  | 12.61 | 0.61 | 11.56 | 1.5 | 10.11 | 1 | 9.01 | 1.08 |
| 12 |  |  | 13.71 | 1.7 | 13.09 | 0.1 | 10.89 | 0.89 | 10.05 | 1.5 |
| 14 |  |  | 14.57 | 1.5 | 14.31 | 1.3 | 12.61 | 1.6 | 11.59 | 1.59 |
